# Bacterial Cellulose Network from Kombucha Fermentation Impregnated with Emulsion-Polymerized Poly(methyl methacrylate) to Form Nanocomposite

**DOI:** 10.3390/polym13040664

**Published:** 2021-02-23

**Authors:** Helena Oliver-Ortega, Shiyu Geng, Francesc Xavier Espinach, Kristiina Oksman, Fabiola Vilaseca

**Affiliations:** 1 Group LEPAMAP, Department of Chemical Engineering, University of Girona, EPS. Ed. PI. C/ Maria Aurelia Capmany 61, 17003 Girona, Spain; helena.oliver@udg.edu; 2 Division of Materials Science, Department of Engineering Sciences and Mathematics, Luleå University of Technology, SE 97187 Luleå, Sweden; shiyu.geng@ltu.se; 3 Design, Development and Product Innovation, Department Organization, Business Management and Product Design, University of Girona, C/ Maria Aurelia Capmany 61, 17003 Girona, Spain; francisco.espinach@udg.edu; 4 Mechanical & Industrial Engineering (MIE), University of Toronto, Toronto, ON M5S 3G8, Canada; 5 Engineering Materials, Industrial and Materials Science, Chalmers University of Technology, SE 41296 Göteborg, Sweden; fabiola.vilaseca@udg.edu; 6 BIMATEC, Department of Chemical Engineering, University of Girona, EPS. Ed. PI. C/ Maria Aurelia Capmany 61, 17003 Girona, Spain

**Keywords:** bacterial cellulose, kombucha fermentation, PMMA, emulsion polymerization, mechanical composites, nanocomposites

## Abstract

The use of bio-based residues is one of the key indicators towards sustainable development goals. In this work, bacterial cellulose, a residue from the fermentation of kombucha tea, was tested as a reinforcing nanofiber network in an emulsion-polymerized poly(methyl methacrylate) (PMMA) matrix. The use of the nanofiber network is facilitating the formation of nanocomposites with well-dispersed nanofibers without using organic solvents or expensive methodologies. Moreover, the bacterial cellulose network structure can serve as a template for the emulsion polymerization of PMMA. The morphology, size, crystallinity, water uptake, and mechanical properties of the kombucha bacterial cellulose (KBC) network were studied. The results showed that KBC nanofibril diameters were ranging between 20–40 nm and the KBC was highly crystalline, >90%. The 3D network was lightweight and porous material, having a density of only 0.014 g/cm^3^. Furthermore, the compressed KBC network had very good mechanical properties, the E-modulus was 8 GPa, and the tensile strength was 172 MPa. The prepared nanocomposites with a KBC concentration of 8 wt.% were translucent with uniform structure confirmed with scanning electron microscopy study, and furthermore, the KBC network was homogeneously impregnated with the PMMA matrix. The mechanical testing of the nanocomposite showed high stiffness compared to the neat PMMA. A simple simulation of the tensile strength was used to understand the limited strain and strength given by the bacterial cellulose network. The excellent properties of the final material demonstrate the capability of a residue of kombucha fermentation as an excellent nanofiber template for use in polymer nanocomposites.

## 1. Introduction

Cellulose is the main structural component of the primary cell wall of most plants but can also be obtained from bacteria [1]. Some bacteria, like *Acetobacter* and *Glucanobacter*, synthesize extracellular polysaccharides such as cellulose to form protective envelopes around the cells [2,3]. These bacterial cellulose nanofibers exhibit very interesting properties such as high purity and crystallinity, with the nanofibrils arranged in interconnected planes as 3D network [4]. Bacterial cellulose is commonly produced from a media based on a mixture of sugars with a certain concentration and pH and temperatures around 3.0 and 28 °C, respectively [5,6]. The process has been adjusted from the synthesis of the Philippine sweet Nata de Coco, which is mainly composed of bacterial cellulose [7]. Nonetheless, there are other processes that have been developed where a pellicle of bacterial cellulose can be obtained, such as the production of kombucha tea. Kombucha tea is a traditional Chinese beverage that later has been produced and consumed worldwide [8,9]. The beverage, known for many positive benefits for human health, is produced from the fermentation of sugars and tea, usually black tea, by a symbiotic culture of bacteria and yeast [10,11,12]. The presence of *Acetobacters* and *Glucanobacters* strains in the culture produces by-products in the shape of kombucha bacterial cellulose pellicles (KBC) [13,14].

However, this by-product is scarcely used, although it mainly consists of a high-quality cellulose nanofiber network. Thus, it could have great potential as reinforcement in polymer-based nanocomposites. In order to meet the objectives of climate change and sustainability, the sustainable development goals to 2030, research interest has grown towards the profit of agriculture residues instead of using fibers such as plant fibers or wood fibers. The use of KBC as reinforcement in cellulose-based nanocomposites represents an improvement for sustainability compared to plant or wood fibers because of the pure cellulose content of the KBC that will not require any chemical treatments or produce extra-residue after treatment [15]. Moreover, its intrinsic structure can achieve one of the milestones in nanocomposites production, the proper dispersion and distribution of the reinforcement along the polymer matrix. The KBC network, with individual nanofibrils in a 3D network, will naturally provide an efficient dispersion of the nanofibers in a polymer matrix. However, despite the apparent benefits of using KBC and bacterial cellulose (BC) membranes as reinforcement in nanocomposites, very often, solvent exchange with the use of organic solvents or supercritical drying are reported in the literature as preparation methods [16,17]. These procedures share known drawbacks in terms of sustainability and preparation costs. A new methodology is required to obtain correct impregnated nanocomposites without the use of organic solvents or an expensive drying process. In this sense, the use of an aqueous polymer solution can improve the diffusion of the polymer through the BC membranes due to the high affinity of BC with water.

Poly(methyl methacrylate) (PMMA) is a synthetic polymer with amorphous structure and high transparency. It also has good tensile and flexural strength, UV tolerance, chemical resistance, and heat resistance. Cellulose/PMMA nanocomposites have been previously prepared showing improvements in stiffness while maintaining the thermal stability of the polymer [18,19]. A reduction of the optical transmittance is generally observed in these nanocomposites due to the nanofiller properties and the presence of aggregations [19,20], and the effect becomes severer by increasing the nanofiller content. In addition, PMMA-based nanocomposites were prepared using solvent exchange methodologies in most studies, which are complex and not environmentally friendly.

In this work, a KBC network was used for the first time as a reinforcement in a PMMA matrix. Furthermore, a novel and environmentally friendly processing method was used to achieve well-dispersed and distributed bacterial cellulose nanocomposites. The process included the impregnation of the KBC using an aqueous emulsion polymerized PMMA. The use of an aqueous solution of the polymer for the direct impregnation instead of solvent exchanges facilitated the impregnation of the KBC network by the polymer while reducing the cost of the process. This method allowed preserving the KBC nanofiber network structure during the PMMA impregnation and drying. To the best of our knowledge, there are no similar studies made before.

Thorough characterization of the KBC network was performed, and later optical, mechanical, thermal, and dynamic mechanical properties of the produced PMMA-KBC nanocomposite were characterized and discussed.

## 2. Materials and Methods

### 2.1. Bacterial Cellulose Production from Kombucha Tea

Kombucha bacterial cellulose (KBC), a residue from kombucha tea fermentation, was used as a raw material in this study. The used KBC was prepared using the following procedure: a mixture of 10 g/L of black tea and 60 g/L glucose were mixed with 4 L of hot water. The symbiotic culture of bacteria and yeast (SCOPY) for the fermentation was added to the solution previously cooled to room temperature. The fermentation was continued for 14 days at room temperature. Thereafter, the fermentation process was finished, and the KBC that formed at the solution surface was removed. The KBC pellicles were thoroughly cleaned in a 0.5 M solution of NaOH at 70 °C for 4 h, in order to remove yeasts and bacteria attached to the cellulose nanofibers. Next, the pellicles were cleaned with hot water (70 °C) for 4 h and then maintained in water until the marron-yellow coloration from tea components disappeared.

For the characterization of the nanofibers, 3D KBC aerogels were prepared by freezing the samples with liquid nitrogen and freeze-drying them with an Alpha 2-4 LD plus freeze-dryer equipment (Martin Christ Freeze Dryer, Gothenburg, Sweden). For mechanical testing of the network, the KBC was dried at 70 °C in a ventilated oven for 48 h. Later, the sample was pressed at 120 °C with 1.3 MPa to remove the water.

### 2.2. PMMA Emulsion Polymerisation

Methyl methacrylate (MMA) monomer from Sigma-Aldrich (Stockholm, Sweden) was polymerized by means of water emulsion polymerization by adding 2.5% (*w/w* of MMA) of potassium peroxidisulphate (Sigma-Aldrich) as initiator and 0.5% (*w/w* of MMA) of docusate sodium salt (Sigma-Aldrich) as an emulsifier. The PMMA emulsion was synthesized following the process described in our previous studies [21,22]. Shortly, the polymerization reaction was performed at 80 °C for 3.5 h. The MMA monomer was slowly added to the mixture, maintaining a continuous and vigorous stirring to keep the PMMA particle size.

### 2.3. Preparation of PMMA-KBC Nanocomposites

**Figure 1:**
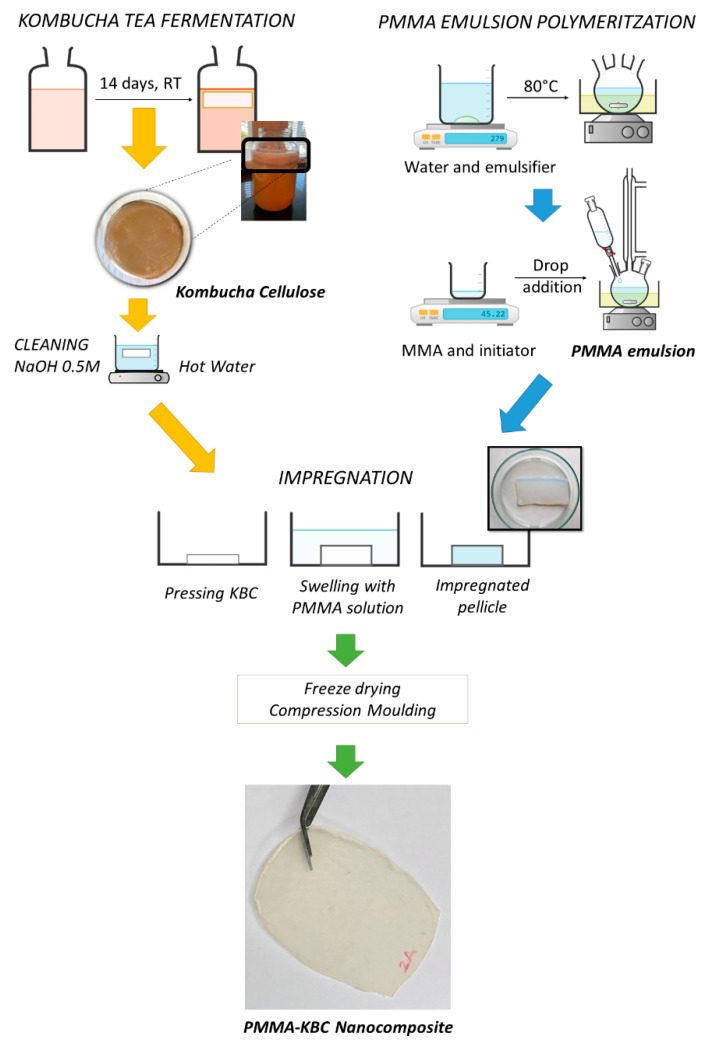
Schematic process of poly(methyl methacrylate)-kombucha bacterial cellulose (PMMA-KBC) nanocomposite production from kombucha tea fermentation and PMMA emulsion polymerization. MMA: methyl methacrylate; RT: room temperature.

PMMA-KBC nanocomposites were obtained by impregnating the KBC network with the PMMA aqueous emulsion. Firstly, the wet KBC was pressed at 0.1 MPa for 3 min to remove excess water and then immersed into the PMMA emulsion. The process was repeated two times. Afterward, the impregnated KBC templates were freeze-dried using the same freeze dryer described above and hot-pressed using a LabEcon 300 press (Fontijne Grotnes, Vlaardingen, the Netherlands) working at 170 °C and 2.5 MPa for 1 min. Nanocomposite films of a thickness of around 300 µm were produced following this process. The KBC content in the films was determined by the final weight of the films and related to the initial weight of the KBC used for the impregnation and its solid content. The average cellulose content in the films was calculated to 8 wt%. Films of pure PMMA were obtained following a similar procedure. PMMA pellets were isolated from drying the PMMA water emulsion and pressed under the same temperature and time conditions. Figure 1 shows the schematic production of PMMA-KBC nanocomposites.

### 2.4. Characterization Methods

Scanning electron microscopy (SEM) was carried out using an FEI Magellan 400 Extreme high resolution—scanning electron microscope (XHR-SEM) (Hillsboro, OR, USA) to study the morphology of the KBC network and PMMA-KBC nanocomposite, as well as to measure the fiber width. The sample surfaces were sputter-coated with platinum using Leica EM ACE200 sputtering instrument (Leica, Wetzlar, Germany) to avoid charging. Nanofiber size (width) was measured from SEM pictures using ImageJ software; the size distribution was based on more than 100 measurements from different images.

The water uptake behavior of KBC aerogel was determined by controlling the weight increment of 0.7 cm^3^ aerogel cubes immersed in water until saturation. The cubes were first dried overnight in an oven at 80 °C.

The crystallinity of the KBC was measured using X-ray diffraction (XRD) PANalytical Empyrean diffractometer (Almelo, The Netherlands) with Cu Kα radiation (λ = 1.5418 Å) and using a 2θ angular range of 5−40°. The crystallinity index (CrI) of KBC was calculated from the following equation [23].

(1) $$CrI\;\left(\%\right)=\;\frac{I_{002}-I_{am}}{I_{002}}\cdot 100$$

where *I*_002_ is the intensity of the peak around 22–23° and *I_am_* is the minimum between the observed peaks, corresponding to the amorphous phase of the cellulose. The crystallite size was determined from the diffractogram using Scherrer’s equation (Equation (2))

(2) $$Crystallite\;size\;=\;\frac{K\lambda }{\beta cos\theta }$$

where *K* is the shape factor with a common value of 0.9, *β* is the peak width at half of its maximum intensity, and *θ* is the Bragg’s angle.

Fourier transformed infrared spectra (FT-IR) of the KBC, PMMA, and PMMA-KBC were obtained by using a Vertex 80v vacuum FT-IR spectrometer (Bruker, Billerica, MA, USA). The equipment was operating in a range between 400 and 4000 cm^−1^.

The mechanical properties of the KBC nanofiber network, PMMA, and PMMA-KBC nanocomposite were determined using a universal tensile testing machine (Shimadzu Autograph AG-X, Kyoto, Japan) equipped with 1 kN load cell and a DVE-101 video extensometer. The gauge length was 20 mm, and the strain rate was 10%/min (2 mm·min^−1^). Sample dimensions for tensile testing were 5 mm in width and 40 mm in length. The thickness was 0.3 mm for the nanocomposite and polymer films and 0.07 mm for the nanofiber network. For statistics, five specimens were tested for each sample.

KBC aerogels cubes with a size of 13 × 4 mm were tested under compression using a Q800 DMTA (dynamic mechanical thermal analyzer, TA Instruments, New Castle, DE, USA) at room temperature with a preload of 0.005 N, an initial strain of −0.5%, and a strain rate of −10%·min^−1^.

Also, the optical transmittance of the neat PMMA and the nanocomposite films (thickness approx. 300 µm) was examined in the visible range (400 to 800 nm) using a Shimadzu UV-Vis 160 spectrophotometer (Shimadzu Europa GmbH, Duisburg, Germany). In addition, a digital camera (Nikon D3000 digital camera) was used to take photos of PMMA and PMMA-KBC nanocomposites used as filters, and the images were processed with Adobe Photoshop software to calculate the changes of the HSL (Hue, Saturation, and Lightness) values when the films are used as optical filters.

The glass transition temperatures of PMMA and PMMA-KBC nanocomposites were studied through differential scanning calorimeter (DSC) using a Q2000 DSC by TA Instruments (New Castle, DE, USA). The samples were heated at a rate of 10 °C·min^−1^ using a flow rate of 40 mL min^−1^ of nitrogen gas as an inert atmosphere. The initial temperature was 30 °C, and the sample was heated to 180 °C.

Dynamic mechanical thermal analysis (DMTA) of PMMA and PMMA-KBC nanocomposite was studied using the Q800 DMA (TA Instruments) in a temperature range of 30–160 °C with a heating rate of 2 °C·min^−1^ and a frequency of 1 Hz.

Finally, the thermal stability of KBC and its PMMA-KBC nanocomposites was studied with a Q500 thermogravimetric analyzer (TA Instruments). The temperature ranged from 25 to 900 °C with a heating rate of 10 °C·min^−1^ and a 60 mL·min^−1^ flow rate of nitrogen as an inert atmosphere.

## 3. Results

### 3.1. Structure and Properties of Kombucha Bacterial Cellulose

**Figure 2:**
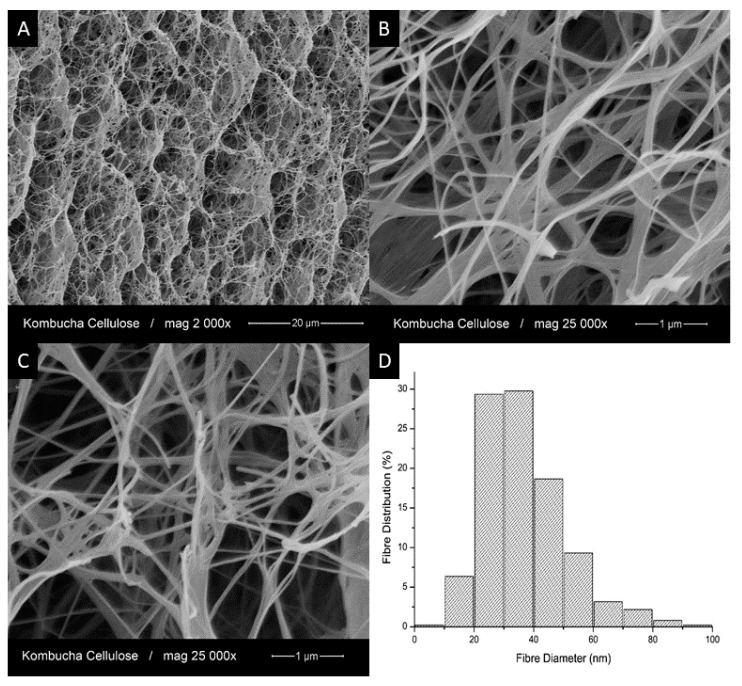
Scanning electron microscopy (SEM) micrographs of the KBC showing (**A**) the general structure of the KBC nanofiber network; (**B**,**C**) its magnified structure details, and (**D**) size distribution of the KBC nanofibers present in the network (width).

The structure of kombucha bacterial cellulose (KBC) and the KBC nanofiber diameter distribution were investigated by scanning electron microscopy (SEM), as shown in Figure 2. At low magnification (Figure 2A), the freeze-dried KBC showed a highly porous structure with different levels of pore size. At higher magnification (Figure 2B,C), both individual nanofibers and some larger fiber bundles could be seen. These fiber bundles were likely formed during the freeze-drying process. The diameter distribution of the KBC nanofibers was obtained from at least 100 measurements. The average diameter was found to be 37 ± 14 nm, with 60% of the nanofibers in the diameter range of 20 and 40 nm (see Figure 2D).

The physical and mechanical properties of KBC in the form of aerogel and nanofiber networks, respectively, are presented in Table 1. The KBC aerogels showed high porosity and very low density due to the high-water holding capacity of the KBC pellicle with only 0.8% of solid content. KBC aerogels can be considered lightweight materials since their density is only 10 times higher than that of normal air (0.0012 g·cm^−3^). The density of the KBC aerogels was similar to that of freeze-dried cellulose nanofibers (CNFs) isolated from biomass at low nanofiber concentrations [24], while they demonstrated much higher stiffness compared to the latter (lower than 100 kPa compression modulus according to the literature) owing to their integral interconnected 3D structure [25,26]. The aerogels also had extraordinary water uptake ability that reached a value of 5079% ± 797%. The KBC nanofiber networks had quite high porosity because the 3D structure of the bacterial cellulose prevents further compaction of the nanofibers during drying and pressing of the KBC pellicle. Other authors also reported low densities for bacterial cellulose networks related to high porosities [1,27]. However, the tensile mechanical properties of the KBC networks were remarkable, with values of ultimate tensile strength higher than that of some nanofiber networks produced from cellulose nanofibers [28], which can be attributed to the interconnected network.

**Figure 3:**
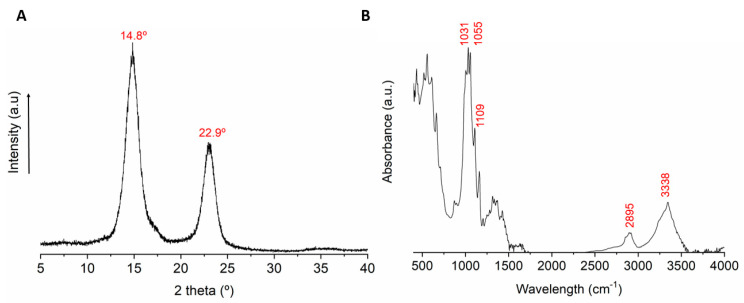
(**A**) X-ray diffraction (XRD) diffractogram of KBC showing the high crystallinity of the sample due to the narrow double peak. (**B**) Fourier transformed infrared (FT-IR) spectrum of KBC with typical bands of pure cellulose.

The original kombucha tea contains several organic compounds that may affect the structure of the bacterial cellulose during the fermentation process. Therefore, in order to determine the degree of crystallinity and chemical structure, X-ray diffraction (XRD) and FT-IR spectroscopy were applied for the produced KBC. Figure 3A presents the XRD pattern of KBC. Two main peaks were observed at 14.8 and 22.9° related to cellulose I [29,30], which were assigned to the (101) and (002) diffraction planes. The crystallinity index of KBC was found to be 89.7%, in the range of the high degree of crystallinity also reported in previous studies [31,32,33]. The crystallite size was calculated from the XRD diffractogram using Scherrer’s equation (Equation (2)). The equation was applied to the most intense peak (2θ = 14.8, plane 101), and the average crystallite size was found to be 5.2 nm, which agrees with earlier reported crystallite size of bacterial cellulose with only small differences regarding the carbon source and the growing process [6].

The chemical structure of KBC was studied using FT-IR spectroscopy (Figure 3B). The broad band at 3338 cm^−1^ relates to the stretching of hydroxyl groups in cellulose, while the small band at 2895 cm^−1^ corresponds to the stretching of the C–H bonds. Small bands in the range of 1400–1200 cm^−1^ belong to C–OH, and C–H bending. The band at 1109 cm^−1^ is assigned to the O-glycosidic bond in cellulose, and the bands at 1055 and 1031 cm^−1^ relate to the stretching of C–OH [34]. Some authors measured the crystallinity index from the FT-IR as the relationship between the absorbance of the rocking C–H band at 1371 cm^−1^ and the absorbance with the highest intensity between 2990 and 4000 cm^−1^ [35]. According to this, a crystallinity index of 0.97 was found for the KBC in this study.

**Figure 4:**
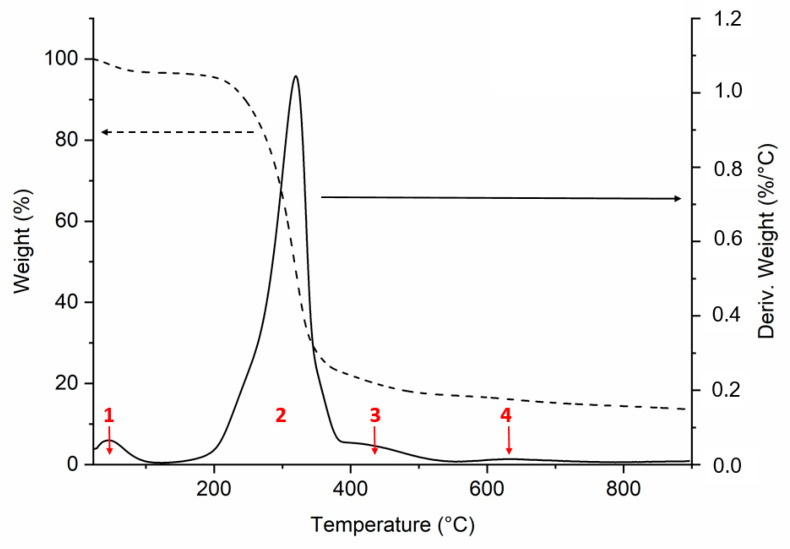
TGA of the KBC, dashed line corresponds to the weight loss; and the black line represents the derivative thermograph. The red numbers correspond following processes: (1) water loss; (2) KBC main degradation; (3) decarboxylation and ring formation reactions, and (4) char formation.

Thermogravimetric analysis (TGA) was performed to determine the thermal stability of KBC (shown in Figure 4). From the thermograph, four weight loss processes were observed. The first one (indicated as 1 in the figure) from 30 to 100 °C was related to the loss of water of the sample. The second one corresponds to the main degradation temperature with an onset temperature of 267 °C, which is consistent with the degradation onset of pure cellulose (270 °C) [36]. The maximum kinetic of this degradation is found at 319 °C. The third degradation step started around 375 °C with the maximum kinetic at 433 °C that can be attributed to decarboxylation and ring formation reactions. The fourth degradation step corresponding to the char transforming process found at approx. 630 °C, where dehydrogenation and condensation reactions occurred [37]. Because the lack of oxygen in the sample resulted in the incomplete conversion of the carbon in cellulose into CO_2_, a residue content of 13.7% was reached at 900 °C. These results are similar to those obtained for other common bacterial cellulose samples [38].

### 3.2. Optical Properties of PMMA and PMMA-KBC Nanocomposites

**Figure 5:**
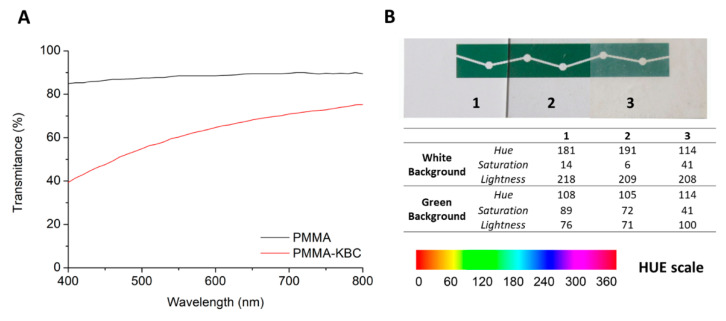
(**A**) VIS-transmittance of PMMA and PMMA-KBC nanocomposites. (**B**) Hue, Saturation, and Lightness (HSL) values of the control (**Left**) and HSL values after using PMMA film (**Middle**) and PMMA-KBC film (**Right**) as filters; the description of the numbers for the Hue scale are included.

The PMMA films had high transparency, whereas the PMMA-KBC nanocomposite films were translucent. The transmittance of the films is presented in Figure 5A. A clear loss of the transmittance was observed for the nanocomposite as compared to the neat polymer. This loss was more noticeable at the low wavelength values, which correspond to the most energetic wavelengths of the visible spectra (VIS).

Variations in the color perception appeared when the PMMA and PMMA-KBC nanocomposite films were used for filtering the wavelengths. The effect was studied using an optical camera to detect changes in the HSL color system (Hue, Saturation, and Lightness) (Figure 5B). Control values were measured over white and green areas on the substrate. The Hue variable relates to color perception and tonality, Saturation refers to the color intensity, and Lightness correlates with secondary colors. The numbers of the Hue scale represent different colors, also shown in Figure 5B. On the white background, the PMMA filter provided a higher Hue value, although it remained in the range of cyan color and decreased the Saturation and Lightness values. The PMMA-KBC filter, instead, reduced the Hue value towards green tonalities while it increased the Saturation value. On the green background, PMMA filter did not make any significant changes for either Hue, Saturation, or Lightness values. However, the PMMA-KBC filter generated considerable changes in the Saturation and Lightness, which implies that some information about the color was lost. The use of the PMMA-KBC nanocomposite as a filter reduced the intensity of the green color. This effect was expected as the transmittance of the nanocomposite was lower than neat PMMA at lower wavelengths (Figure 5A). The results confirmed that PMMA-KBC nanocomposites could act as a filter altering color perception, indicating their potential concerning optical applications.

### 3.3. Mechanical Properties of the PMMA-KBC Nanocomposites

The mechanical properties of the neat PMMA and the PMMA-KBC nanocomposite obtained from tensile testing and are compared in Table 2. Noticeably, the Young’s modulus of the nanocomposite was almost twice as high as that of the neat PMMA, indicating that the addition of just 8 wt.% of KBC had a significant effect on improving the stiffness of the PMMA. The rigidity of the 3D structure of the KBC, together with the good dispersion and distribution of the nanofibers along the polymer matrix, can explain the stiffening effect observed for the PMMA-KBC nanocomposite. However, the tensile strength and the elongation at break of the nanocomposite were lower than those of the neat PMMA. The possible reason is that the strong and rigid KBC network limited the flexibility of the nanocomposite and led to breakage at an earlier stage.

**Figure 6:**
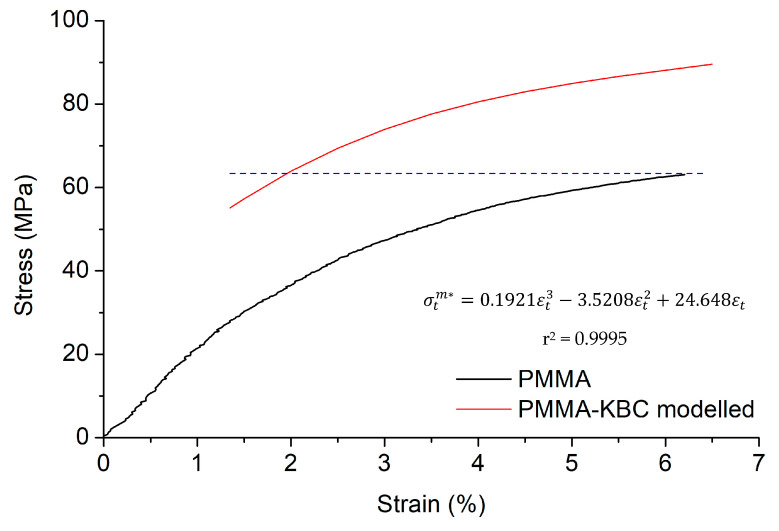
PMMA experimental curve and PMMA-KBC modeled curve obtained from the simulation of the experimental results in the rule of mixtures (ROM). The blue dash line indicates the maximum strength of PMMA polymer matrix.

To further investigate the strength of the PMMA-KBC nanocomposite, simple model was applied according to the experimental results. In Figure 6, a representation of the stress-strain curve of the neat PMMA is presented, together with the polynomial regression equation ($\sigma_{t}^{m*}=0.1921\varepsilon_{t}^{3}-3.5208\varepsilon_{t}^{2}+24.648\varepsilon_{t}$) that relates the stress of the PMMA matrix (*σ_t_*^m*^) with its deformation (*ε_t_*) in percentage. From this regression, the tensile strength of the nanocomposite at 1.4% strain (55.1 MPa) was significantly above the strength value of the neat polymer at the same strain (27.3 MPa) according to the regression equation. Therefore, the addition of KBC largely improved the strength of the polymer at this strain. At this point, by the use of the rule of mixtures (ROM) (Equation (3)) one can estimate the theoretical intrinsic tensile strength of the KBC reinforcement.

(3) $$\sigma_{t}^{C}=\sigma_{t}^{F}\cdot V^{F}+\sigma_{t}^{m*}\cdot \left(1-V^{F}\right)$$

where *V^F^* refers to the fiber volume fraction, and *σ_t_^C^* and *σ_t_^F^* to the tensile strength of the composite and KBC reinforcement, respectively. The value of PMMA’s contribution is computed from the regression curve of the experimental values. Using the ROM (Equation (3)) at the maximum strength and deformation of the composite, the theoretical tensile strength for the intrinsic resistance of the KBC network was determined to be at least 461.5 MPa. This strength is in the range of lignocellulosic fibers or below and not expected for cellulose nanofibers [39,40,41]. Nonetheless, KBC cannot be described as discrete cellulose nanofibers but as a continuous phase formed by 3D network of nanofibers.

The contribution of KBC network to the tensile strength of the nanocomposite corresponds to the first term of Equation (3) (*σ_t_^F^* · *V^F^*), which was 29.5 MPa in the current case. It is worth noting that the contribution of KBC is constant at a defined volume fraction, whereas the contribution of the matrix increases with the strain. Therefore, using Equation (3) and the regression line of the strain-stress curve of the matrix, it was possible to determine the minimum strain where the strength of the nanocomposite will equal tensile strength of the polymer being *ε* = 1.9%. Thus, if the elongation at break of the nanocomposite would be above 1.9%, the material would show higher tensile strength than the matrix. The higher the strain, the higher the increase. Figure 6 shows the expected tensile strength of the composite assuming strains at break between the obtained 1.4% and 6.5%. The figure envisions the strengthening potential of KBC as a reinforcing material. Therefore, more research is needed to find higher strains at break for KBC reinforced materials by preventing a premature failure of the nanocomposite.

The elongation at break of our PMMA-KBC nanocomposite was lower contrasted with that of other PMMA nanocomposites reinforced with cellulosic nanofibers reported in the literature, even at higher reinforcing contents [18,19,42,43]. In order to understand this phenomenon, the failure mechanism of our material needs to be further studied. The low elongation at break can be attributed to crack propagation in the brittle structure. Although PMMA polymer is a ductile material, KBC shows a brittle behavior. It is known that brittle materials catastrophically fail by a single crack propagation [44]. Additionally, KBC structure can act as a crack inhibitor, but the presence of the PMMA matrix as a continuous phase connecting the KBC’s structure can ease the crack propagation. This phenomenon could be stronger than the interface obtained between KBC and PMMA. Indeed, a strong interface will promote a plain crack propagation along with the matrix and the KBC, breaking down the nanofiber structure and preventing any pull-out mechanism.

### 3.4. Morphology and Chemical Structure of the PMMA-KBC Nanocomposites

**Figure 7:**
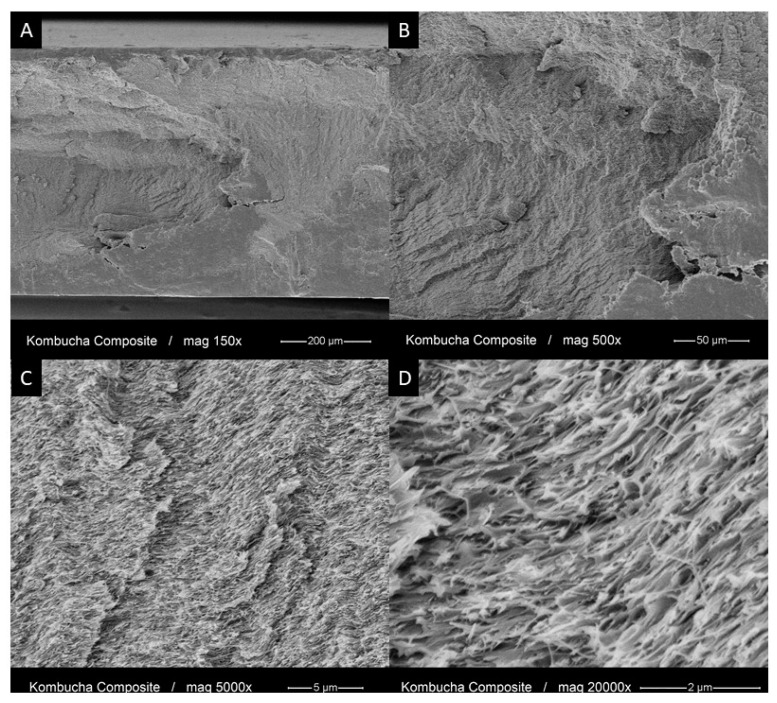
SEM images of the fracture cross-section of the PMMA-KBC nanocomposite at different magnifications. (**A**,**B**) overview micrographs showing brittle fracture of the nanocomposite. (**C**,**D**) detailed views of the nanocomposites with the visible fibrils and intact KBC network at the cross-section.

The morphology of the fractured cross-section of the nanocomposite was studied using higher resolution SEM (Figure 7). The micrographs at lower magnifications (Figure 7A,B) exhibited a brittle fracture surface, which corresponded with their mechanical testing results. Furthermore, the observation of the KBC network at higher magnifications (Figure 7C,D) showed a clear visible KBC nanofibril structure. The 3D structure of the KBC was intact and completely impregnated by the polymer matrix creating a compact structure without apparent voids nor pulled-out fibers. This confirms that the KBC structure was preserved during the production of the PMMA-KBC nanocomposite. The fact that the KBC 3D structure was preserved after material failure demonstrates the brittle failure behavior of our PMMA-KBC nanocomposite.

**Figure 8:**
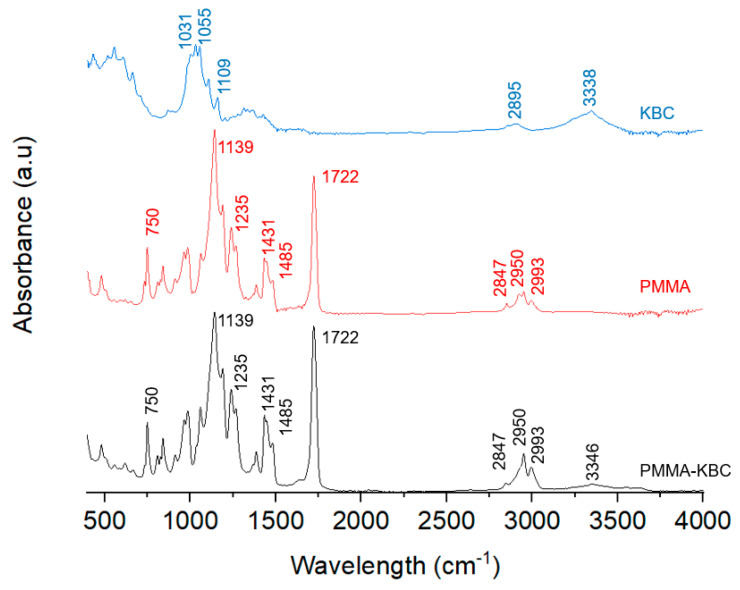
FT-IR spectra of PMMA-KBC (black), PMMA (red), and KBC (blue) indicating the main bands for each of them.

In Figure 8, FT-IR spectra of the PMMA-KBC nanocomposite were compared with that of the neat PMMA and KBC and show some differences. The main bands in PMMA spectrum are also present in the PMMA-KBC nanocomposite; these bands were stretching of carbonyl at 1722 cm^−1^, stretching and bending of methyl groups (at 2950, 2997 cm^−1^ respectively at 1431 and 1485 cm^−1^), C–H stretching, and rocking at 2847 cm^−1^ 750 cm^−1^ and C–O–C stretching at 1139 and 1235 cm^−1^ [20,45,46]. However, it is possible to see that the intensity of the band at 1722 cm^−1^ is increased in the nanocomposite, which likely indicates hydrogen bonds were formed [47,48]. The presence of the KBC in the nanocomposite is confirmed by a weak band at 3346 cm^−1^ in the PMMA-KBC spectrum corresponding to the hydroxyl groups of KBC. The low intensity of the KBC bands in the PMMA-KBC nanocomposites was due to the low concentration (8 wt.%) of the KBC in the final formulation. In addition, a small displacement of the hydroxyl group signal of KBC from 3338 to 3346 cm^−1^ is observed. The shift of the band could also be related to hydrogen bonding interaction between the hydroxyl groups in the KBC and PMMA [49].

### 3.5. Dynamical Mechanical Thermal Properties of Nanocomposites

**Figure 9:**
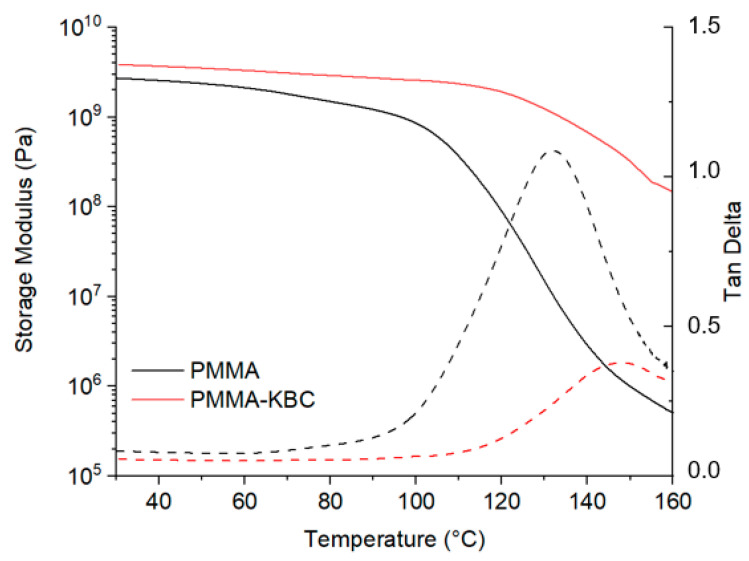
Dynamic mechanical thermal analysis (DMTA) of neat PMMA polymer and PMMA-KBC nanocomposite, storage modulus solid line, and tan δ dashed line as a function of temperature.

Dynamic mechanical thermal properties of the PMMA and the PMMA-KBC nanocomposite are shown in Figure 9. An obvious increase in the storage modulus could be seen for the PMMA-KBC nanocomposite compared to the neat PMMA in the whole temperature range. The reason for this is that the KBC network was restricting the molecular mobility of the PMMA chains leading to higher stiffness of the nanocomposite. The plateau of the storage modulus of the nanocomposite maintained until approx. 120 °C, while that of the neat PMMA started to dramatically decline from approx. 100 °C. The tan δ peak was at 132 °C for the neat PMMA and shifted to 149 °C for the PMMA-KBC nanocomposite, which indicates an interaction between the PMMA and KBC network. The reduction of the PMMA chain mobility likely arises from both the mechanical restriction effect of the KBC network and a favorable interface between KBC nanofibers and the PMMA matrix.

### 3.6. Thermal Properties of Nanocomposites

**Figure 10:**
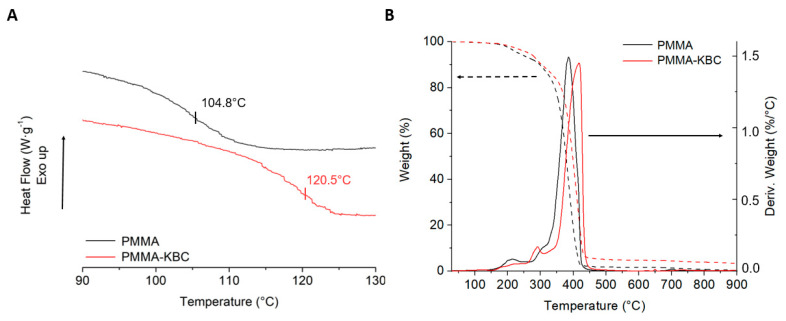
Thermal properties of PMMA and PMMA-KBC nanocomposites. (**A**) DSC thermograph magnification of the T_g_ of neat PMMA and PMMA-KBC nanocomposite; (**B**) TGA weight loss (dash line) and weight loss rate (continuous) curves of the samples.

To determine the thermal properties, DSC and TGA were performed for the samples (Figure 10). The neat PMMA polymer is a completely amorphous material with a glass transition temperature (T_g_) of 104.8 °C (Figure 10A). This result agrees with the values found in the literature for emulsion polymerized PMMA [19,43]. The T_g_ of the PMMA in the nanocomposite shifted to a higher temperature, and this increase is confirming that the PMMA-KBC nanocomposite can serve at a wider temperature range that is beneficial for many applications. Figure 10B illustrates the thermal stability of the neat PMMA and the nanocomposite studied by TGA. The first degradation peak appeared below 200 °C for the PMMA sample, corresponding to the volatilization of some free monomers or oligomers and the breakage of weak head-head linkages produced during the radical polymerization [50,51,52]. For the nanocomposite, this peak was partially shifted to a higher temperature around 290 °C caused by the presence of the KBC network. Moreover, the temperature that the maximum weight loss occurred at increased from 386 °C for the PMMA to 418 °C for the nanocomposite, and the residue content of the nanocomposite was higher. These also indicate that the presence of the KBC network improved the thermal stability of PMMA, likely arising from the well-interconnected network structure of the KBC hindering the emission of the formed volatiles during the degradation. The other possible reason is that cellulose may capture free radicals and consequently prevents polymer degradation. This effect has been observed for other polymer matrices reinforced with cellulosic fibers [45,53,54,55].

### 3.7. Future Perspectives

From the current research work, the next challenges and research perspectives are found in promoting the growth of the PMMA polymer from the kombucha cellulose nanofiber. Using the kombucha cellulose network as a template during the polymer synthesis would facilitate obtaining better deformability and achieve a higher tensile strength of the nanocomposite compared to the net polymer matrix.

## 4. Conclusions

PMMA was successfully reinforced with a kombucha bacterial cellulose network, a residue obtained from kombucha tea fermentation. This residue was characterized and later used as reinforcement. The kombucha bacterial cellulose (KBC) pellicles consisted of a nanofibrous porous network with an average nanofibril diameter of 37 nm. These thin nanofibers assembled in a 3D network are forming a porous structure with excellent mechanical properties. Moreover, the KBC nanofibers have high purity and crystallinity, and their thermal stability is comparable to bacterial cellulose from other resources.

Nanocomposite materials were obtained by impregnating the KBC network with an aqueous emulsion of PMMA. After impregnation, the KBCs were freeze-dried and compression molded to obtain thin nanocomposite films comprising 8 wt.% of KBC. The optical properties showed a loss of the transmittance for the nanocomposite material at low wavelengths. The incorporation of the 3D bacterial cellulose network into the polymer matrix doubled the elastic modulus of the PMMA. This contrasted to a slight reduction of the tensile strength compared to the neat matrix. This phenomenon was attributed to crack propagation in the rigid 3D nanofiber structure, which was also explained by the low deformation of the nanocomposite. The brittle fracture was confirmed by SEM analysis of the fracture surfaces. A facile simulation using the ROM (rule of mixture) demonstrated the strengthening capacity of KBC, with a modeled intrinsic strength of 461.5 MPa. The stiffening effect of the KBC network was also confirmed by dynamic mechanical analysis. Nanocomposites with tan delta peak position shifted towards higher temperature is indicating that the KBC is restricting the molecular relaxation of the PMMA and improving the thermal stability PMMA-KBC nanocomposite. The presence of the KBC network prevented free radicals from faster degradation of the polymer chains, as demonstrated by thermogravimetric analysis.

To summarize, the use of the residue from kombucha fermentation was demonstrated as efficient reinforcement in PMMA, resulting in nanocomposites with high stiffness, interesting optical properties, and improved thermal stability. More research needs to be conducted to avoid premature failure of the nanocomposite responsible for the limited nanocomposite deformation and the low strength of the material.

## Figures and Tables

**Table 1:** Physical and mechanical properties of the KBC aerogels and KBC nanofiber network (* compression test; ** tensile test).

Property	KBC Aerogel	KBC Nanofiber Network
Density (g·cm^−3^)	0.014 ± 0.002	0.807 ± 0.146
Porosity (%)	99.0 ± 0.2	44.5 ± 8.5
Strength	* 227 ± 63 kPa	** 172 ± 24 MPa
Stiffness	* 153 ± 22 * kPa	** 8.0 ± 1.9 GPa

**Table 2:** Mechanical properties of neat PMMA and PMMA-KBC nanocomposite.

Sample	Young’s Modulus (GPa)	Tensile Strength (MPa)	Elongation at Break (%)
PMMA	2.1 ± 0.1	63.4 ± 1.2	6.4 ± 0.7
PMMA-KBC	4.0 ± 0.2	55.1 ± 0.7	1.4 ± 0.1

## Data Availability

The data presented in this study are available on request from the corresponding author.
